# Assessing the cascading impacts of natural disasters in a multi-layer behavioral network framework

**DOI:** 10.1038/s41598-021-99343-4

**Published:** 2021-10-11

**Authors:** Asjad Naqvi, Irene Monasterolo

**Affiliations:** 1 International Institute for Applied Systems Analysis (IIASA), Laxenburg, Austria; 2 Vienna University of Economics and Business (WU), Vienna, Austria; 3 Global Development Policy Center, Boston University (BU), Boston, USA

**Keywords:** Climate-change impacts, Projection and prediction

## Abstract

Natural disasters negatively impact regions and exacerbate socioeconomic vulnerabilities. While the direct impacts of natural disasters are well understood, the channels through which these shocks spread to non-affected regions, still represents an open research question. In this paper we propose modelling socioeconomic systems as spatially-explicit, multi-layer behavioral networks, where the interplay of supply-side production, and demand-side consumption decisions, can help us understand how climate shocks cascade. We apply this modelling framework to analyze the spatial-temporal evolution of vulnerability following a negative food-production shock in one part of an agriculture-dependent economy. Simulation results show that vulnerability is cyclical, and its distribution critically depends on the network density and distance from the epicenter of the shock. We also introduce a new multi-layer measure, the Vulnerability Rank (*VRank*), which synthesizes various location-level risks into a single index. This framework can help design policies, aimed to better understand, effectively respond, and build resilience to natural disasters. This is particularly important for poorer regions, where response time is critical and financial resources are limited.

## Introduction

Natural disasters can give rise to large-scale shocks that disrupt socioeconomic systems through two channels. On the one hand, are supply-side adjustments that result from localized production losses which can lead to trade disruptions^1–4^. On the other hand, are demand-side adjustments that emerge as a consequence of households’ jobs and income losses, resulting in displacement and migration decisions^5–8^. The demand and supply channels interact and co-evolve, potentially amplifying the impact of the shock, and spreading it to non-affected locations^9,10^.

Strong negative feedback effects and non-linear transitions in the aftermath of a natural disaster can also result from hitting certain thresholds, such as large economic losses, that can lead to behavior changes^11–14^. For instance, if income levels in disaster-hit locations suddenly fall, and basic consumption needs cannot be met, then households will adjust their behavior to ensure consumption smoothing by running down savings, selling assets, borrowing, or even migrating^6,15,16^. A large population exodus from affected locations will potentially result in non-trivial labor and goods market adjustments in both affected and non-affected locations. In order to respond to these changing market signals, firms will need to adapt production decisions and reevaluate target locations for selling goods. This can also feedback on household decisions causing further non-linearities. These supply and demand-side adaptive decisions will continue to co-evolve until some post-shock equilibrium trends are achieved. While direct impacts of natural disasters have been long studied^2,7^, the analysis of the conditions for indirect impacts to emerge and the magnitude of cascading socioeconomic losses, deserves research attention.

Understanding disaster shocks’ transmission and amplification, and the role of decision-making processes, is crucial for modeling the non-linear spatial-temporal transitions in disaster-affected regions. This, in turn, is important to avoid underestimating risks, and to inform effective policies aimed to build resilience, while climate-induced natural disasters are on the rise and expected to increase^17^. This is particularly relevant in low-income regions where formal social safety nets are weak, governments’ fiscal space is limited, and the timing of policy response is critical^18^.

In this paper, we introduce a modelling framework to assess the indirect impacts and cascading disasters’ losses. We develop a spatially-explicit agent-based multi-layer network model, where supply-side production and trade decisions^19^, and demand-side labor supply and migration decisions, interact, based on agent-based behavioral rules derived from the literature.

Our modelling framework allows us to integrate the supply and the demand-side dimensions, that are usually analyzed separately^20–22^, in order to obtain a more robust assessment of cascading effects following natural disasters. The bottom-up agent-based rules, that define the interactions both across and within network layers, result in meso- and macro outcomes that again feedback on micro-level decisions resulting in complex non-linear interactions^18,23^. In order to reduce this complexity, we introduce a multi-layer measure, the Vulnerability Rank, or *VRank*, which synthesizes various dimensions of risk into a single index. The *VRank* builds on recent advances in multi-layer network measures^24–26^, where high-risk nodes in a network are identified based on their own properties and of their neighbors. In particular, our *VRank*, inspired from the *DebtRank*^27^, analyzes vulnerability propagation in socioeconomic multi-layer networks by looking at the accessibility and affordability of a minimum consumption bundle of each location nodes’ network cluster.

We set up the multi-layer network model on an agriculture-dependent region, where we introduce an exogenous food production shock in one part of the network. Then, we assess how the shock cascades to the non-affected part of the network through bottom-up demand and supply-side behavioral interactions. In the model, we track spatial-temporal distributive effects through price and consumption volatility. Finally, we estimate the *VRank*, to identify the highly vulnerable locations in the post-shock transition phase. Simulation results show that vulnerability is cyclical, and its distribution depends critically on the network density and distance from the epicenter of the shock.

The remaining paper is organized as follows. “Literature review” reviews state of the art literature. “The multi-layer behavioral network framework” presents the conceptual framework for setting up a multi-layer model for assessing the impacts of scenarios of natural disasters. “Model description” presents the multi-layer agent-based model and the *VRank*. “Assessing the impacts of a natural disaster on food trade networks” discusses the results of the simulations and “Conclusions and research steps ahead” concludes, highlighting the policy relevance of the results in the context of disaster risk reduction, and discussing avenues for future research.

## Literature review

There is consensus among scientists that climate change will results in higher incidences of both sudden and slow-onset natural disasters^8,17^ that will invariably affect global production systems especially agriculture^28–30^. This aspect, coupled with a growing share of population living in areas exposed to climate change challenges the resilience of the global food system^31^. If agriculture-dependent areas, especially bread-basket regions, that supply the bulk of essential food items, are impacted by natural disasters, it can trigger strong negative impacts across food trade networks^29,32^. Since bread-basket regions also employ a large share of the working population at the regional level, usually at very low wages, a climate-induced disaster (for example, a flood) could create conditions for food insecurity in the directly-impacted locations. Additionally, this can also spillover to other locations through trade and migration networks causing cascading losses^28,33^. Understanding how the distributional impacts of natural disasters evolve is crucial for assessing socioeconomic vulnerability and to inform policy response^11,23^.

In this regard, network models provide a unique tool to identify and model socioeconomic interactions. In the past two decades, network models have gained significant attention for the analysis of complex socioeconomic interactions in coupled human-environmental systems, especially in relation to measuring resilience^34–36^. This is due to their ability to incorporate behavioral aspects that requires dealing with economic complexity, thus going beyond the scope of standard optimization-based modeling tools. Network models have been applied to a diverse array of topics like complex product spaces and economic growth^37,38^, technological innovation and diffusion^39^, migration flows^40,41^, urban sprawl^42^, urban mobility^43^, supply-chain networks^1,19^, resilience in financial networks^27,44^, climate insurance^45^, and climate stress tests^46,47^. The development of network models and their application has been supported by better data availability, access to higher computational power, and interest in understanding the deeply inter-connected world from a complex network lens.

More recently, multi-layer or multi-dimensional networks^25,48^ have received considerable attention, in particular after the 2008 global financial crisis^46^. In multi-layer networks, complexity not only plays a role within, but also across different network layers that usually interact through price signals. For example, in the field of finance, recent literature has analyzed the interaction of different asset classes and financial institutions in multi-layer settings. Network models have also contributed to the analysis of systemic risk, where under specific conditions, a shock to a relatively benign part of a network layer could cause a contagion through interconnectedness, potentially leading to large-scale losses or even a complete system collapse^44,47,49^.

Network models have recently been applied to the analysis of climate-related financial risks as well, for example, in climate stress tests^47,50^. Climate stress tests allow us to understand the conditions for risk amplification and reverberation of a low-carbon transition in a complex network setting, thus providing a comprehensive assessment of risks and opportunities of various climate-finance related policies^46^.

In the context of disaster risk assessment, several papers have applied variations of the multi-layer network model presented in this paper. For example, applications include modeling impact of the 2010 Indus Basin floods^51^, analyzing the spatial spillover effects of the 2005 Kashmir earthquake^13^, and combining multi-layer networks with copulas to assess how the 2003 droughts in India could have potentially compounded the risks due to correlated changes in weather patterns^33^.

Overall, the importance of complexity approaches for the analysis of the socioeconomic impacts of climate change has been increasingly recognized^52^. This line of thinking is driven by factors like fat-tailed distributions of the nature of climate impacts^52^, non-linearity between climate impacts and heterogeneous socioeconomic agents’ reactions^23^, decision-making under uncertainty^53^, feedback loops across agents and sectors, the timing and path dependency of policy responses^23^, and more recently, the interaction of climate change with the financial system^54^. Nevertheless, bridging the linkages between supply-side networks (such as production and trade) and demand-side networks (such as consumption and migration) is still in an infant stage^18,55^.

For analyzing the indirect impacts and cascading effects of natural disasters, the integration of multi-layer networks with agent-based models (ABM) allows us to overcome the limitations of traditional economic models that solve to equilibrium and embed representative agents with perfect foresight and forward-looking expectations (that is, it assumes that agents have perfect knowledge of the future), and usually focus on long-run aggregate outcomes like output growth and capital stocks^52^. Such models do not allow us to assess indirect and cascading impacts of natural disasters, and most importantly, the post-shock transition phase. ABMs depart from the assumptions of representative agents and rational expectations^56^ and instead analyse agents’ heterogeneity, adaptive expectations, and bounded rationality, and focus on decision-making under uncertainty^23^. Since ABM are a bottom-up methodology where micro interactions generate “meso” and macro outcomes, which in turn, feedback on the micro decisions the models can easily handle non-linear transition processes^23^. This allows us to account for heterogeneous spatial and temporal preferences, asymmetric information, and path-dependent outcomes in a natural disaster-like shock scenario where we can simulate the evolution of vulnerability hotspots^23^.

## The multi-layer behavioral network framework

**Figure 1:**
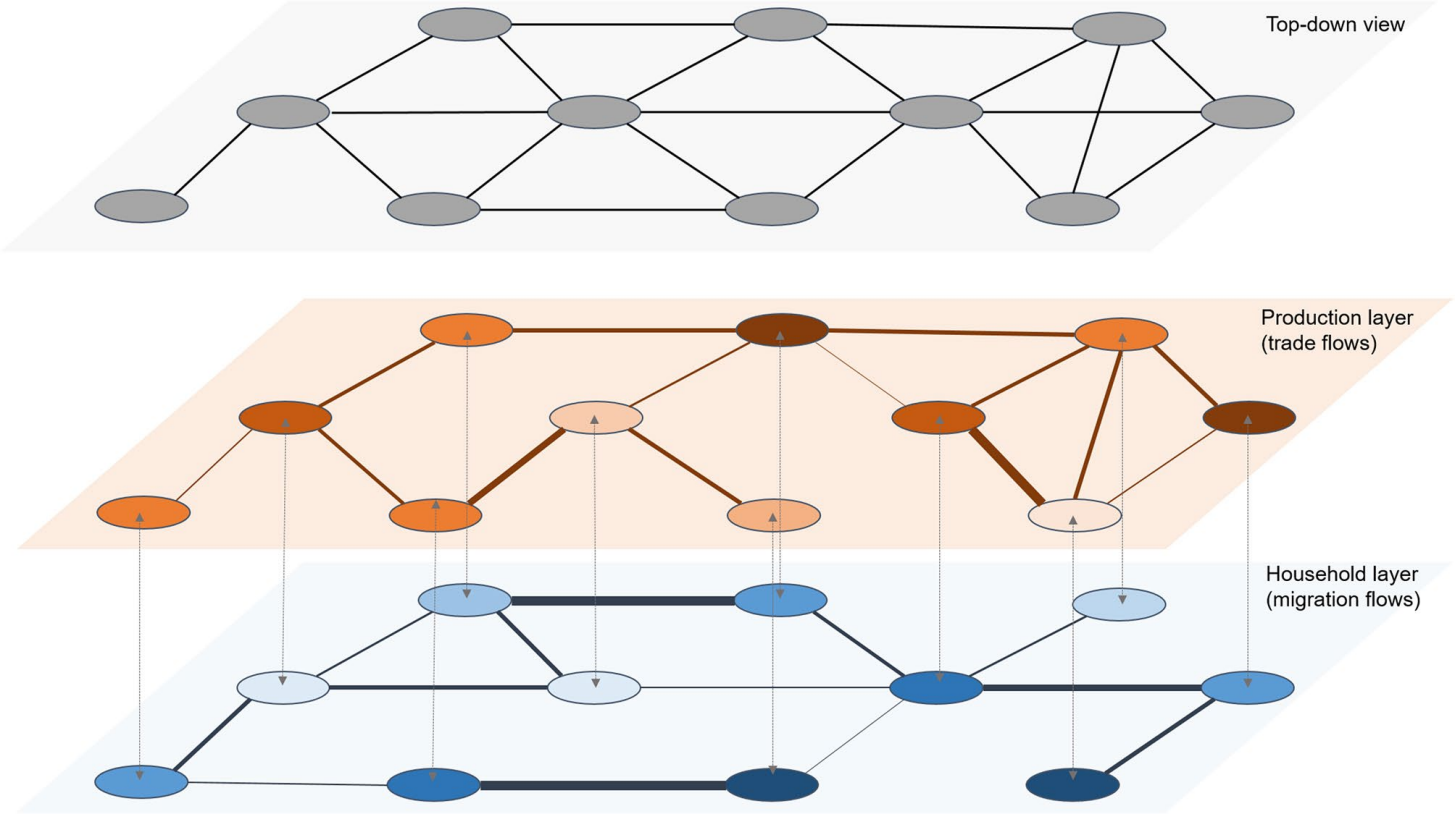
A stylised multi-layer network framework. The figure shows the Production and Household layers which are integrated in a socioeconomic network. The same network nodes belong to both layers, but the inter-connectivity might vary by layer. The nodes interact with each other through behavioral rules both within and across layers. In this framework, a shock to one node in one layer, for example, a food output loss, will cascade across the network through the different layers.

In Fig. 1, we represent a stylized economy composed of multiple locations that are split across two network layers: (i) a production layer, which determines how much is produced and traded based on price signals, and (ii), a household layer, which determines labor supply and migration levels determined by wage signals. The two layers also feedback across each other, for example household determine the demand for goods, while the demand for goods generates labor demand.

In the figure, the location nodes are connected to other nodes with different intensities across the two layers. This implies that certain locations might be more central in one network than in another. These differences have strong implications for exogenous shocks. For example, if a node, that is central for food distribution in one network layer faces a catastrophic shock, then it will have strong impacts not only in the production layer but also in the household layer since it will affect the supply and prices of goods in neighboring nodes. In turn, this will cause a decline in purchasing power triggering further negative consequences along the network as the shock cascades. This is a main difference from traditional networks observed from a top-down perspective, where all flows across layers are aggregated together, usually in monetary terms (for example in Input–Output tables).

**Figure 2:**
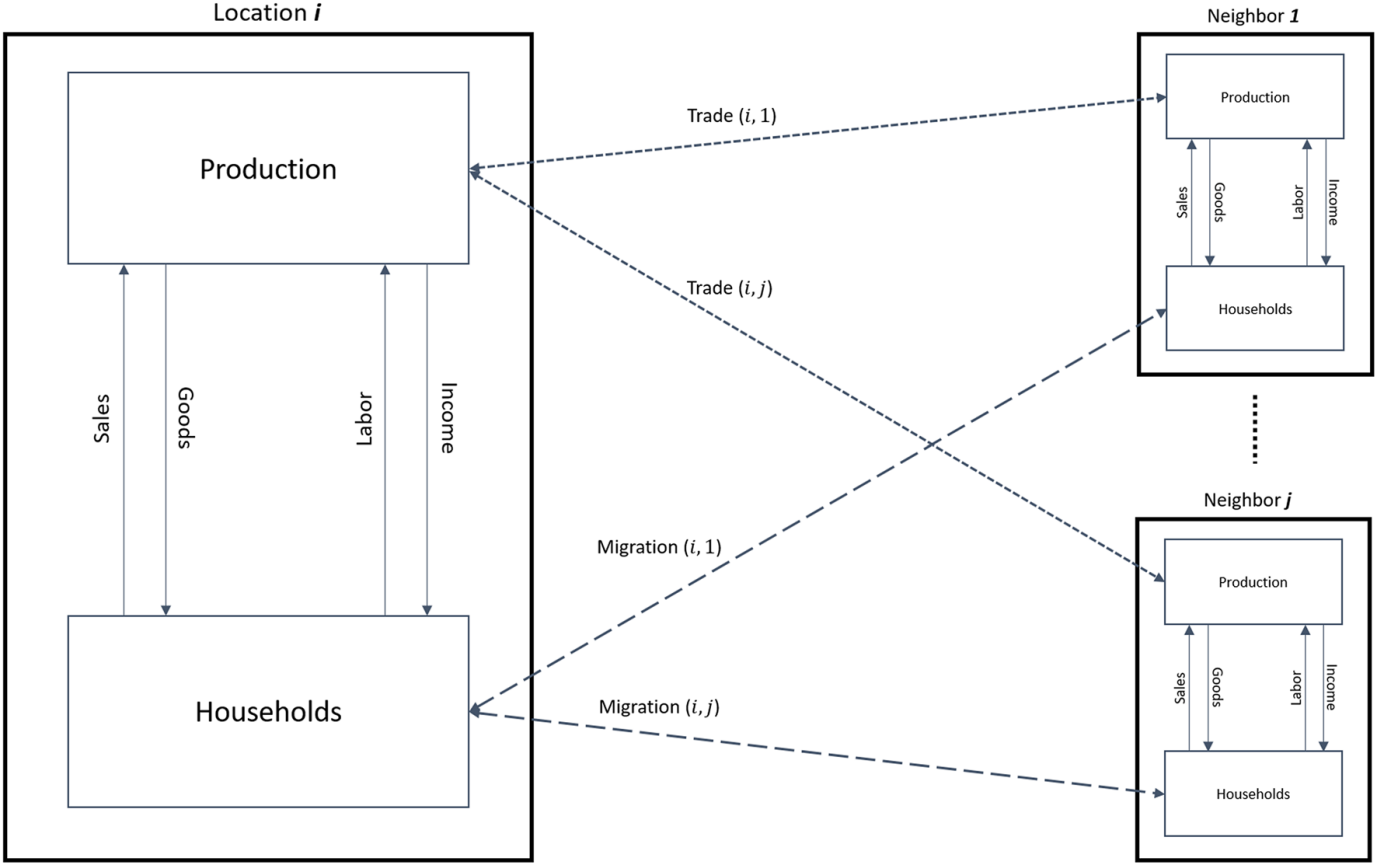
Behavioural interactions across location pairs. This figure shows the behavioral interactions between location pairs. Each location node follows a circular economy framework where endowments of labor and capital determine income and production levels. Differences in relative prices and wages across a location *i* and its *j* neighbors result in migration and trade flows in a gravity model-like setting.

In order to operationalize trade and migration flows, the nodes in the multi-layer network need to be endowed with decision-making behavioral rules. Figure 2 displays stylized economic interactions across different locations. Each location node *i* owns a stock of capital and labor that characterize the production and household layers respectively. At each node *i*, labor is employed to produce an output in exchange for wages, which is then used to purchase and consume goods. Thus each location forms a circular flow economy evolving its own set of market wages and goods prices. A node *i* also interacts with its *j* neighbors. Since the neighbors also have their own stocks of labor and capital, they have their own circular flow dynamics resulting in their location-specific market wages and prices. Heterogeneity across the location nodes will therefore imply differences in prices. Due to these differences, the interaction of node *i* with its *j* neighbors will result in trade and migration flows, where trade is driven by relative price signals and migration by relative wage signals. Here we assume the standard economic case where higher good prices and higher wages attract higher goods and labor flows in a gravity model-like setting^6,57^. These flows will continue until there is an equalization of prices eventually resulting in a stable distribution of labor and goods flows across the system.

### The impact of a natural disaster on a food-trade network

**Figure 3:**
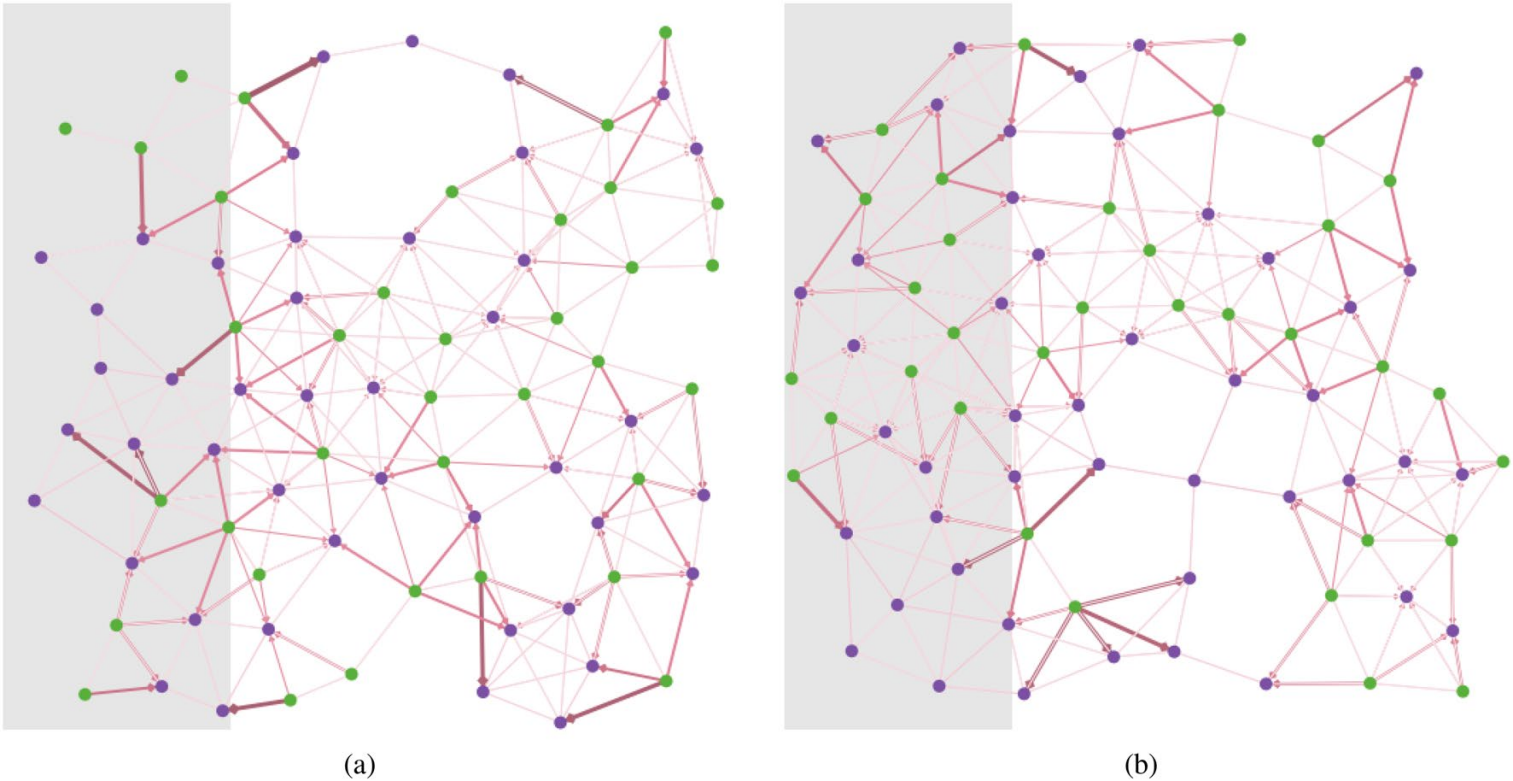
Sample of two random multi-layer networks. The networks are composed of food producing (green) and non-food producing (purple) nodes, with link weights representing the level of food flows. The shaded area represents the epicenter of a natural disaster.

We consider an economy that produces two types of goods, basic food commodities (for example, crops like rice or wheat) and non-food commodities and services. We assume, for simplicity, that different locations have different endowments of these goods allowing for trade of goods and services as shown in two randomly generated networks in Fig. 3.

Let us consider a natural disaster, for example a flood, which affects the locations in the grey zone, the epicenter of the shock in Fig. 3. The flood destroys agricultural output, thus reducing food supply. In the model setup, this shock will immediately result in two key changes in the epicenter region; (i) a sudden rise in food prices due to lower output, and (ii), a fall in wages, due to lower production and employment. As a result, behavioral responses to the shock will unfold in the production and household layers. Households in the affected locations respond to the shock by moving to non-affected neighbors which offers relatively higher incomes and thus higher purchasing power. However, the increase in population in non-affected locations reduces wages, since output is not rising in the very short run, thus forcing population displacement even further away from the epicenter.

The demographic shift also affects the demand for goods. As agents moves from affected to non-affected locations, the demand for goods also shifts more towards the non-affected locations. Goods producers, observing higher profits in non-affected locations, redirect their supplies out of the disaster-affected nodes. This results in further shortages in the affected locations, leading to price increases and reducing the purchasing power of households there. On the one hand, it fosters more migration away from the epicenter. On the other hand, producers in affected locations can now sell in more profitable markets and thus they increase wages to attract more workers, slowing down the migration. The interaction of the two demand and supply layers continues through market signals, resulting in reinforcing and balancing mechanisms until the labor and goods prices converge to some post-shock equilibrium.

### Economic thresholds

**Figure 4:**
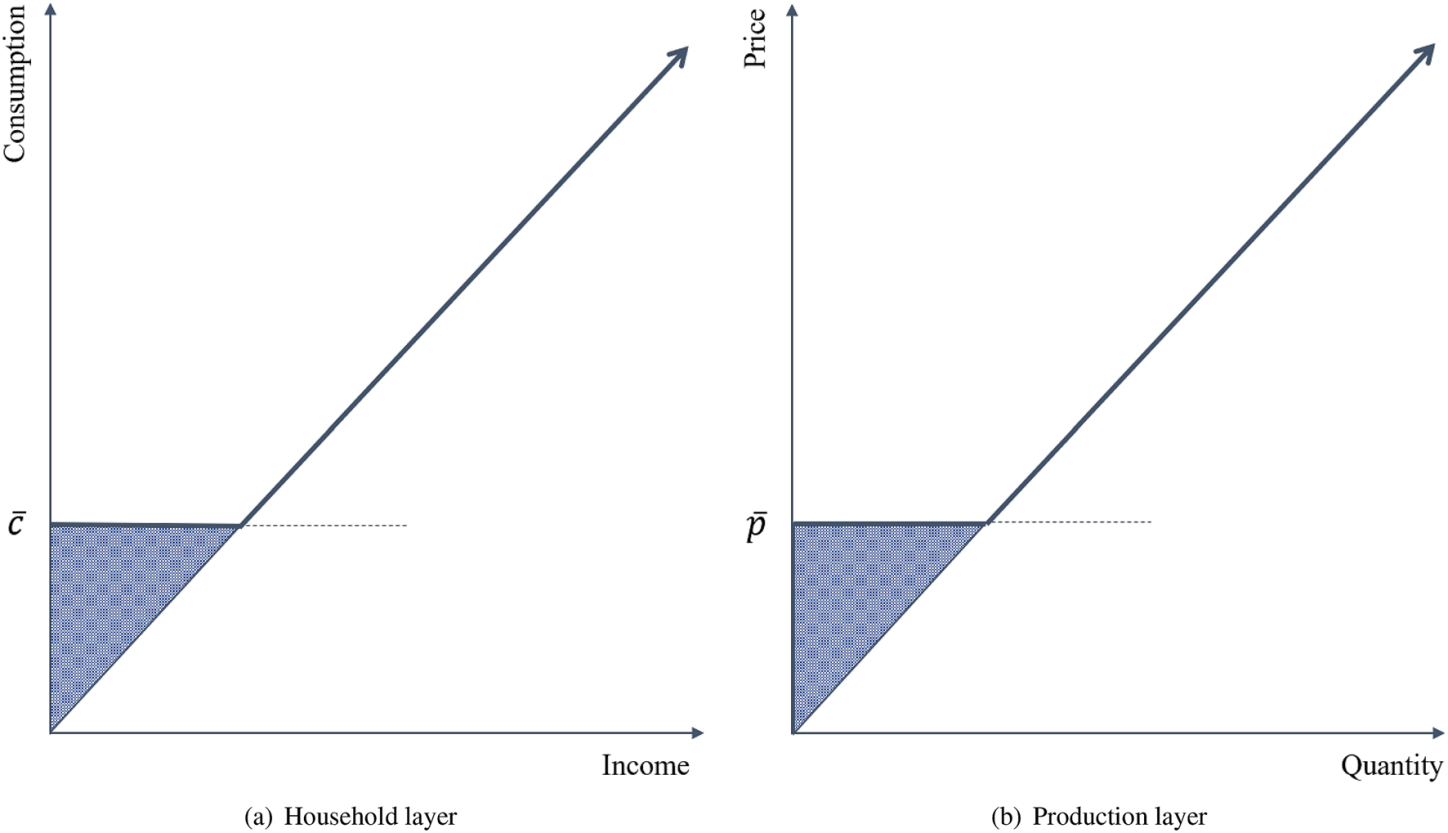
Thresholds in a multi-layer behavioral network. The figure shows how the thresholds look like for the household and production layers. Households falling below minimum consumption level $\bar{c}$, introduce consumption-smoothing strategies and migrate. Likewise, firms stop selling through the markets, where market price is below cost price $\bar{p}$.

Responses of agents to the direct and indirect shocks can lead to thresholds that result in behavioral regime switches that change the patterns of interactions. Figure 4 shows the two thresholds incorporated in the model.

Let us consider again our food trade network composed of two layers. If food is subject to inelastic demand, as is the case for basic food consumption items in low-income regions, then a price increase implies that households will spend a higher share of their income on food to stay above some minimum consumption threshold (Fig. 4a). Falling below this threshold would trigger income-consumption smoothing strategies. For example, running down savings and food inventories^58^, selling of assets^59^, and informal and formal borrowing mechanisms^60,61^. If all else fails, households migrate to other locations to avoid the risk of becoming “food insecure”^62,63^. This, in turn, could give rise to new socioeconomic vulnerabilities in the destination locations.

To keep the model at a manageable level of complexity, we do not add household stocks of food or savings but we assume that migration is the only coping mechanism. This solution gives the same qualitative results without adding additional complexity of managing stocks, which has been analysed in Refs.^13,51^.

Similar to the household layer, the production layer also faces thresholds. Producers sell goods only in the locations where they can make profits or, at least, where they can cover the cost of production (Fig. 4b). This, for example, suggests that markets impacted by the shock, which have high production costs and low demand (for example, as a result of out-migration) are likely to be less attractive destinations for selling goods. Thus, by changing the supply, profit-seeking producers can affect market prices, which feed back on households’ consumption and migration decisions. The combination of these two thresholds can significantly increase the non-linearity in the post-shock transition phase.

## Model description

The core model structure assumes full adjustment of demand and supply through price signals across the goods and labor markets. These assumptions can be easily modified to incorporate frictions, institutional barriers, and path-dependencies that will lead to stronger negative impact of the shock^6,13^. Therefore, the aim here is to show that even in the best-case full-adjustment scenario one can observe a heterogeneous evolution of vulnerability.

A multi-layer network is defined by $i=\{1,\dots ,n\}$ nodes. Each node *i* is endowed with land and capital stock that results in the production of food (*F*) and other non-food items (*G*) respectively. The production set is represented by $k=\{F,G\}$ and the output of each good equal $x_k$. The total output produced at a node *i* equals:

1 $$\begin{aligned} y_i=\sum _k{\beta _k x_{ik}}, \end{aligned}$$

where $\beta _k$ is the weight of product *k* at node *i*. 50% of randomly selected nodes mostly produce food ($\beta _F=0.9, \beta _G=0.1$), while the rest mostly produce mostly other goods ($\beta _G=0.9, \beta _F=0.1$). For simplicity, all nodes are endowed with the same level of production capacity and productivity levels, such that real output, $y_i$, is equal across all nodes. In our setup, the variations are randomly generated network structures. However, this structure can be further expanded to include a basket of goods across different nodes.

The production at node *i* employs all the available labor $L_i$. Assuming that $\rho$ is the unit cost of production and labor is the only cost (capital is assumed fixed in the short-run), the income per worker equals:

2 $$\begin{aligned} w_i=\frac{\rho y_i}{L_i}. \end{aligned}$$

The income earned by workers is fully spent on the the two goods as follows: a fraction $\alpha$ is spent on food *F*, and $1-\alpha$ on good *G*. From here, the price of *F* and *G* at node *i* can be derived as:

3 $$\begin{aligned} p_{iF}= & {} \frac{\alpha w_i L_i}{x_{iF}} \nonumber \\ p_{iG}= & {} \frac{(1-\alpha )w_i L_i}{x_{iG}}, \end{aligned}$$

where the average price index of each node is the weighted sum of the goods over the total number of goods:

4 $$\begin{aligned} p_i = \frac{\sum_{k=1}^K{\gamma_k p_i^k}}{K} = \frac{p^F_i + p^G_i}{2}. \end{aligned}$$

We assume that both the goods *F* and *G* have equal weights, or $\gamma _F=\gamma _G=1$. In nominal terms, the value of the total output produced at a node *i* can be derived as:

5 $$\begin{aligned} Y_i=\sum _k{x_{ik} p_{ik}}. \end{aligned}$$

### Trade and migration

Nodes are fully allowed to interact with connected neighbors through a diffusion process. A gravity model-like specification determines how much migration and trade takes place across the layers^41,55,62^. This procedure is defined as follows. At each time tick, the node has priors about its own stock of labor and goods, and evaluates the price and wage signals of its connected neighbors. The set of destination locations with a net positive gain are ranked based on the extent of the positive gain from trade or income. A pre-determined fraction $0 < \mu _i \le 1$ is diffused to the target destinations based on the relative shares calculated from the distribution of the gains. The amount diffused can also vary across the layers, for example, by assuming that goods adjust faster than labor flows. For the sake of simplicity, the diffusion rate is kept homogeneous across the household and production layers. The value of $\mu$ does not impact overall results but slows down the transition process from one equilibrium to another in the model.

The diffusion value $\Pi _{ij}$ is calculated using a joint-probability distribution function of the following form:

6 $$\begin{aligned} \Pi _{ij} = \Pi _{ij}^q \times \left( 1-\Pi _{ij}^d \right) . \end{aligned}$$

**Figure 5:**
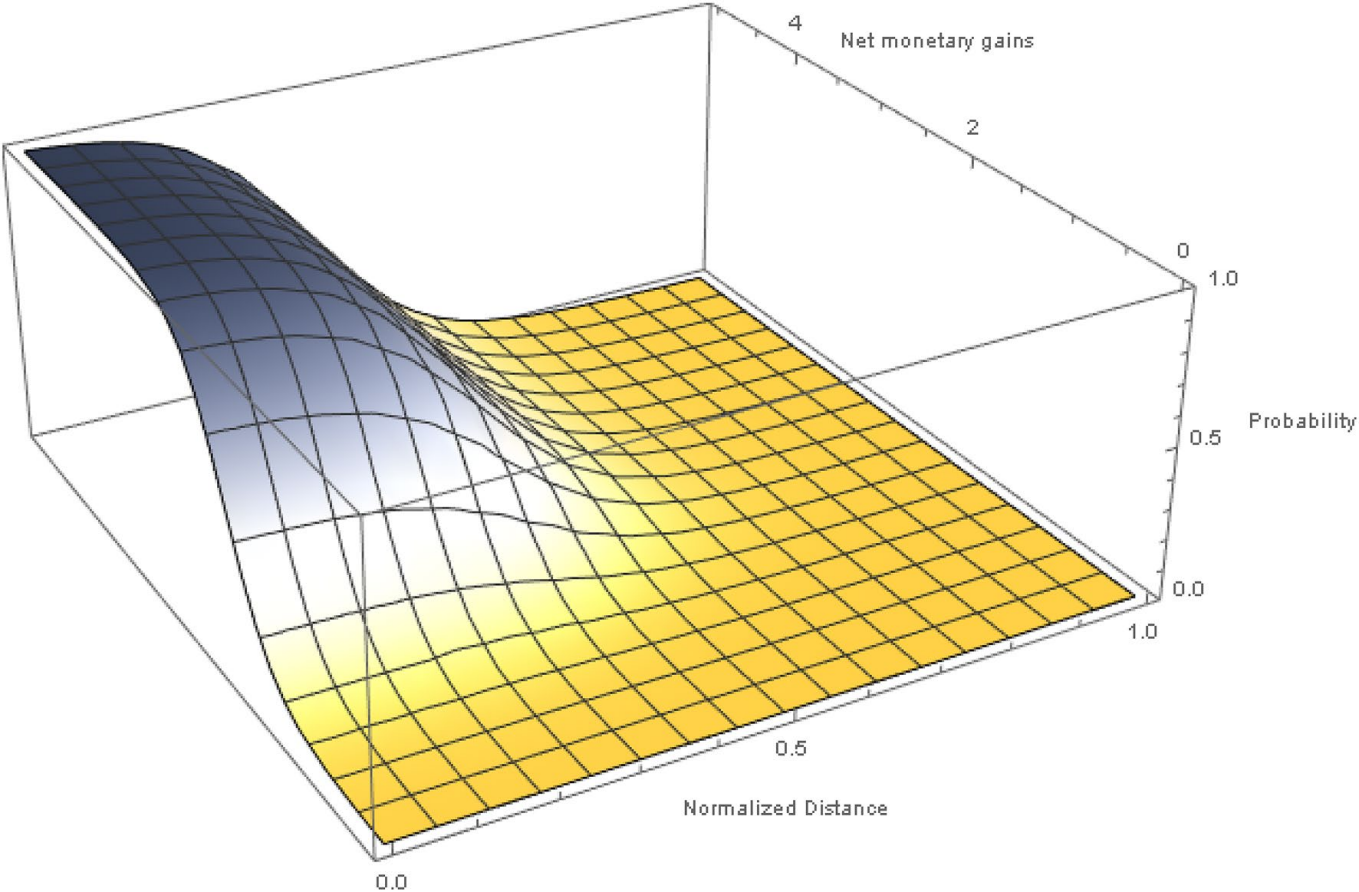
Probability density distribution for flow decisions. In our model, the net monetary gain between a target node and the current node has a positive weight on migration or trade decisions, while distances have a negative weight.

According to Eq. (6), the probability of migrating or selling to a neighbor *j*, $\Pi _{ij}$, is positively affected by relative economic gains $\Pi _{ij}^q$, and negatively by distance $\Pi _{ij}^d$. Here distance can be tangible or intangible costs of migration or transporting a good to a destination. For migration, economic gains are determined by real income differences and for trade by relative profit gains. Distance enters in the model as a normalized function between zero and one such that the farthest away point from a location *i* has a probability close to zero. Distance is not directly monetized in the model, for example, in terms of travel or transport costs, but acts as a counter-balance to always selecting destinations just based on purely economic gains. Figure 5 shows the space of the joint-probability distribution, which also highlights the distance versus economic gain trade-off. This implies that there could be closer locations with a small economic gain that have an equal weight to farther-away locations with a higher economic gain.

Probabilities are estimated using a generic logistic function:

7 $$\begin{aligned} \Pi ^z = \frac{1}{\left( a+be^{-z}\right) }, \end{aligned}$$

where $\{a,b\}$ are parameters that take some reasonable values to allow for a smooth transition between the extreme points. The logistic function gives a probability distribution between zero and one, where a small net gain gives probability values close to zero and if the gains are very high the probabilities are close to one. For normalized distances, the probabilities are reversed such that the closest nodes have a probability close to one, or the distance-based penalizing factor is almost zero.

Each node calculates a vector of probabilities for its connected neighbors where the probabilities are normalized to sum up to up to one to determine the share of quantity diffused to each node. For example, a node with four neighbors estimates a probability vector of $\{0.2, 0.5, 0.7, 0.7\}$ where the last two locations are highly desirable and have equal weights. Since the sum of this vector is 2.1, the normalized share comes out to $\{0.10, 0.24, 0.33, 0.33\}$ which adds up to one. Therefore the first location gets 10% of the diffused value, while the last two nodes get 33% each. The key concept here is that locations with higher net gains pull a higher share of the quantity available for diffusion. The decision to select a location is deliberately not made binary, where the highest-gain node would get everything as would be the case in a utility maximization setting with optimal solutions. Here we allow agents to probabilistically choose a location, and therefore allow agents to make mistakes by choosing the next-best options, as long as the gains are net positive^13^. Additional probability distributions can also be added to represent other factors such as trade linkages, community at destination, and barriers to mobility like border controls.

### Thresholds

The thresholds discussed earlier are formalized in the model as follows:

8 $$\begin{aligned} \alpha = \mathbf{Max} \left[ \frac{p_{i}^F \bar{c}}{w_i L_i}, \bar{\alpha } w_i L_i \right] . \end{aligned}$$

According to Eq. (8), households adjust their propensity to consume food relative to a minimum consumption value $\bar{c}$. If their income decreases or food prices increase, then $\alpha$ will adjust endogenously to ensure that they stay at least at the $\bar{c}$ level. Households cannot spend more than what they earn, and they have to consume at least $\bar{\alpha }$, where $\alpha$ is bounded such that $\bar{\alpha } \le \alpha \le 1$. Producers only sell in markets which give them positive profits. Producers can diversify their portfolio to sell their output in neigboring markets, adjusting their supply to changes in relative prices. Since producers have a production costs associated with outputs, the difference between the cost and the market price determines the profit margin. Assuming that labor is the only input in the production process, whose unit cost is $\rho$, and the market price equals $p_{ik}$, then the condition $p_{ik} \ge \rho$, defines the criteria for generating a vector of desirable destinations. This vector is sorted based on the profit margins which determines the amount diffused. The logic is similar to migration decisions, where the vector of profit margins is normalized to one to estimate the supply shares to target destinations. If some markets fall below the minimum cost threshold, where $p_{ik} < \rho$, then they are excluded from the destination set. These two thresholds evolve dynamically, and are the key driving mechanism behind shock cascades in the model. They also enable wages and prices to equalize across all the nodes.

### The *VRank* multi-layer risk measure

We introduce a new multi-layer risk measure that we call “Vulnerability Rank”, or *VRank*, to quantify the stress in the system as a result of a natural disaster. Our *VRank* measure is based on recent most relevant advances in multi-dimensional network measures^26,38^. In particular, it abstracts from the *DebtRank* measure of financial distress propagation^27^. *DebtRank*^49,64^, and other multi-layer risk measures are increasingly being applied to better understand systemic risk in financial networks^65^ including in the context of climate-financial risk assessment^47,66^.

By introducing the multi-layer risk measures, we aim to bring the complexity of vulnerability, which arises from various interactions, into a single measure. For example, *DebtRank*^27^ considers the portfolio holdings of banks, their debts and loans and derives a measure of risk of default. Since banks are in a network setting, one bank’s risk is not only determined by its own balance sheets but also those of connected banks that own its debt or lend it money. In this setup, even a small benign node failing can cascade across the whole system potentially resulting in a system collapse. While multi-layer networks and systemic risk measures are becoming more important in the banking sector especially in central banks (for example, the European Central Bank (ECB)^67^), the insights from these applications also have an important, yet under-explored, applications to climate shocks and their subsequent cascading impact on socioeconomic systems.

In the model framework presented here, vulnerability, particularly in low-income food-producing regions, is defined in terms of a minimum consumption bundle ($\bar{c}$ in Fig. 4). Agents falling below $\bar{c}$ are labeled as “food insecure”, and if their consumption needs are not immediately met, it can result in second round negative impacts like poor health, lower productivity, reduced economic output, conflict, and displacement. Minimum consumption bundles are well-defined and usually measured in adult-equivalent caloric in-take per capita, which also determines food-based poverty lines^31^.

In our model setting, the ability to purchase a minimum consumption bundle $\bar{c}$, is determined by price (supply) and income and savings (demand) including the ability to import the supply from neighboring nodes, and earn income elsewhere. Formally, *VRank* is defined as:

9 $$\begin{aligned} VRank_{it} = \left( \frac{p_{it} \bar{c}}{w_{it} L_{it}} \beta \frac{\sum _{j \ne i}{p_{jt}\bar{c}}}{\sum _{j \ne i}{w_{jt} L_{jt}}} \right) ^{1/2}, \end{aligned}$$

where $p_{it} \bar{c}$ is the price of minimum consumption bundle at node *i* at time *t*, $w_{it} L_{it}$ is total income calculated as wage rate at node *i* times the total labor supply. Similarly $p_{jt}\bar{c}$ and $w_{jt} L_{jt}$ are the average minimum consumption bundle to income ratio of the *j* neighboring nodes of node *i*, and $\beta$ is the dampening factor usually set at $\beta =0.85$^68^. In other words, the vulnerability of a node *i* is not only defined as the purchasing power of the minimum consumption bundle of agents at a node location *i*, but also as the average of all its neighboring *j* nodes. A higher value of $VRank_{it}$ implies higher vulnerability.

The *VRank* formula, captures two key elements. First, it shows the dependence of a node from another one in terms of food supply. For instance, a node which does not produce any food commodity would be considered vulnerable in isolation, but if it is connected with several food producing locations that could supply it with food, it would show a much lower *VRank* index. In addition, this node would be in a better position than a completely isolated food-producing node. Second, *VRank* captures the displacement and migration channels of vulnerability. A node that produces enough food for its own consumption might experience a sudden population influx from highly vulnerable neighboring nodes, thus becoming vulnerable to the shock. Therefore, the *VRank* allows us to quantify multiple sources of vulnerability, and also resilience, in a single quantitative index.

## Assessing the impacts of a natural disaster on food trade networks

A stylized model of food trade is developed based on the multi-layer behavioral network framework described above. To simulate the model, we generate random networks with 80 nodes each, of which 50% produces food while the rest produce other non-food consumption goods. Figure 3 shows two sample random networks. All nodes have the same initial population of households and quantities of food and other goods, and exactly the same behavioral rules. These assumptions can be relaxed. However, this will increase the complexity and make it difficult to disentangle the changes in outcomes that result from the shock, as opposed to adjustments resulting from the interaction of heterogeneous agents. The nodes are connected through three sets of links that represent population, food, and other goods flows.

Each network runs till it reaches a stable distribution of population, goods, and prices through migration and trade. The food-producing nodes in one part of the network, shown as the grey region in Fig. 3 are shocked as a consequence of the natural disaster resulting in a reduction in food output. The shock impact ranges from 40 to 80% of output reduction in steps of 10% each. Each shock level is tested on 10 randomly generated networks, or 50 simulations runs in total, to generate distributions. The model runs until goods and labor prices stabilize.

**Figure 6:**
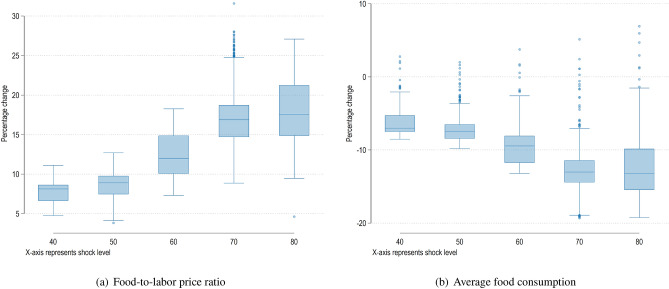
Indirect macroeconomic and distributive effects of a natural disaster in a two-layers food network. In each figure, the x-axis represents different shock levels, while the y-axis shows changes relative to the no-shock baseline scenario. Box plots show medians (box), inter-quartile ranges (lines), and outliers (dots), for multiple simulation runs within the same shock level.

Figure 6 analyzes the indirect impacts of different levels of the disaster shock on two economic indicators as percentage change from the baseline (no-shock) scenario. Figure 6a shows an increase in the relative food-to-labor price ratio. This indicator captures the dual impact of rising food prices and lowering of incomes indicating a decline in affordability. Figure 6b shows average food consumption per capita. As expected, higher shock levels results in an overall decline in consumption levels. Overall, we can also observe that higher magnitude shocks create higher volatility in the system, resulting in stronger distributive effects.

**Figure 7:**
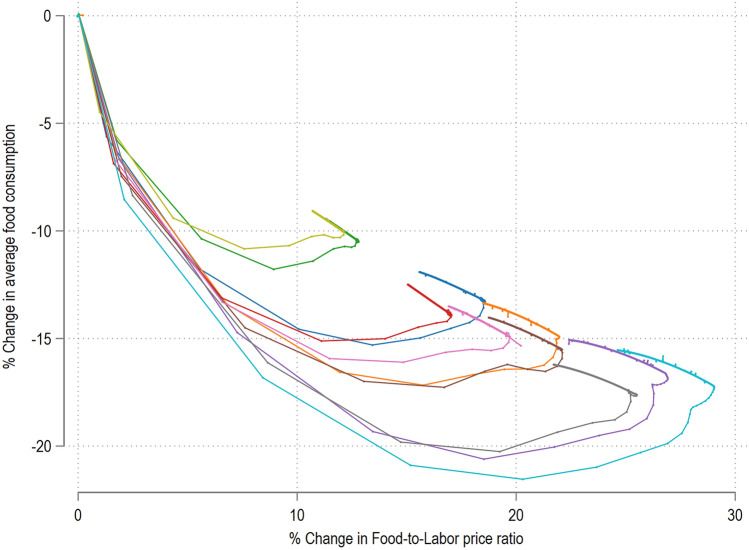
Cyclical Vulnerability for a 80% food shock. The top left corner represents the start of the simulation and the cyclical adjustment takes place in a counter-clockwise direction.

Figure 7 plots the two indicators discussed above and shows the output for ten different simulation runs for the 80% food output shock. We notice the emergence of different cycles in the adjustment phase, leading to two main considerations. First, the structure of the network determines the evolving pattern of the cycles, for example, the numbers of nodes affected, the pace of connectivity across layers and their relative response times. Therefore, the same shock can lead to very different patterns even in presence of similar baseline socioeconomic structures. Second, if we focus only on the starting point (top-left of Fig. 7) and on the last stationary point at the end of the cycle, as is the case in long-period equilibrium models, we would miss the adjustment pathways of the climate-led shocks. Here, larger cycles imply that households experience higher degrees of vulnerability in the transition phase. This result shows the importance of policy response, especially in poorer areas where such cycles can further exacerbate existing vulnerabilities including food insecurity^31,69^.

**Figure 8:**
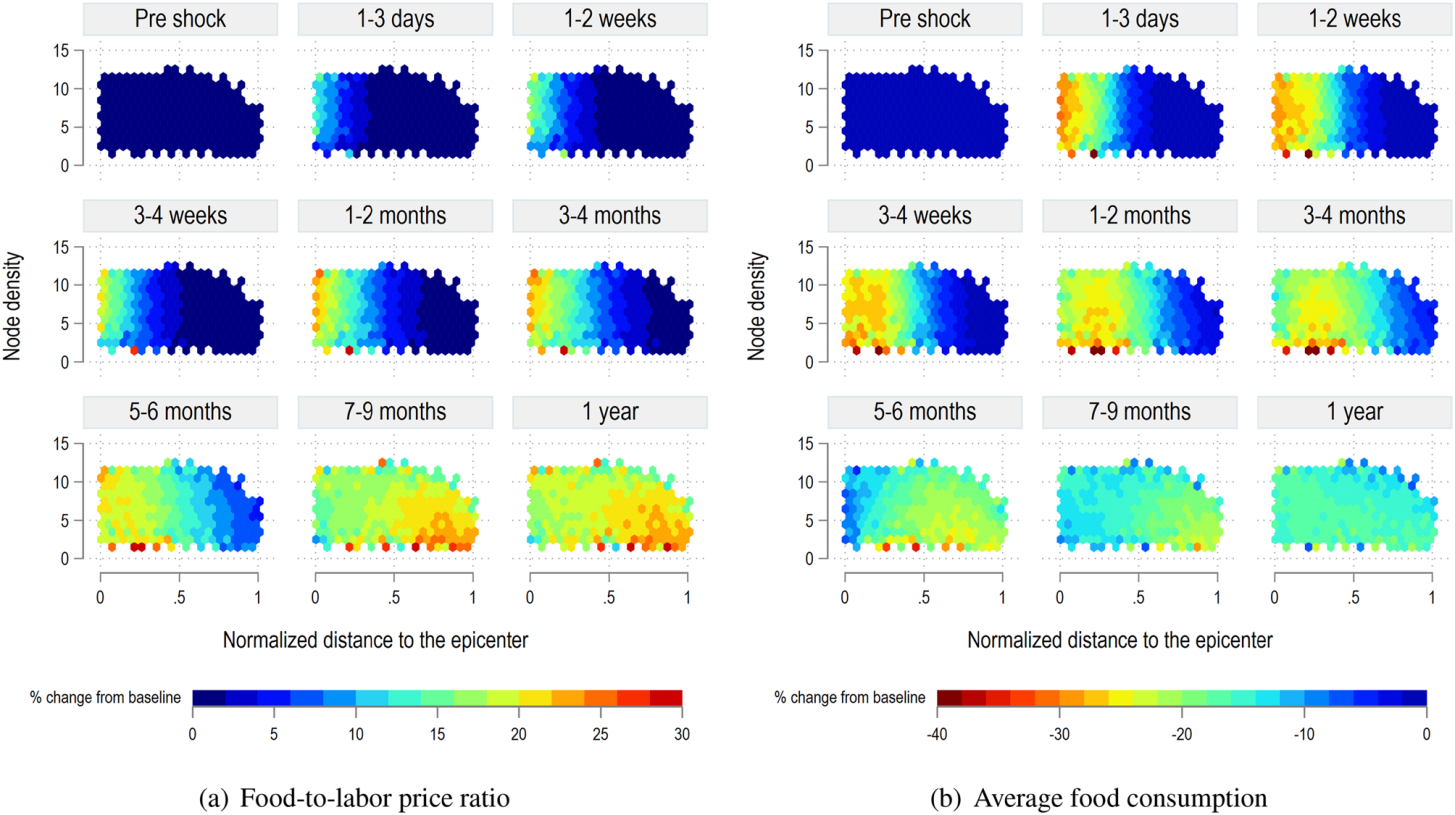
Spatial-temporal distributional impacts of a food output shock.

Figure 8 shows the distributive impacts of the 80% food output shock. The figure shows binned heat plots of the two indicators, the relative food-to-labor price ratio (Fig. 8a), and the average consumption of households (Fig. 8b). The x-axis represents the normalized distance of the nodes from the shocked locations. On this axis, zero represents the epicenter of the shock and one represents the locations farthest away from the epicenter. The y-axis represents the density of the nodes, where a low number implies fewer network linkages. The spatial evolution of average food-to-labor prices and average food consumption indicators is tracked at regular intervals from the time the shock hits the system. In both the figures, the high-density nodes closest to the epicenter respond faster to the shock than the low-density ones. Higher density also implies that the shock is immediately passed to the neighboring nodes where the response time of low-density nodes is much slower. This slow adjustment in low density nodes can be identified as the wave that moves along the x-axis in both the plots shown in Fig. 8.

Figure 8 also highlights the importance of the spatial layout of a region in assessing the impact of the shock. The shock cascades from the top left corner (high density, nearest to the epicenter), to the bottom-right (low density, farthest from the epicenter). The stable post-shock distribution shown in the last time step in Fig. 8 implies that after all the adjustments have stabilized, lower density nodes, especially those farthest away from the epicenter, end up with poorer access to food markets and thus generally show lower food consumption levels.

**Figure 9:**
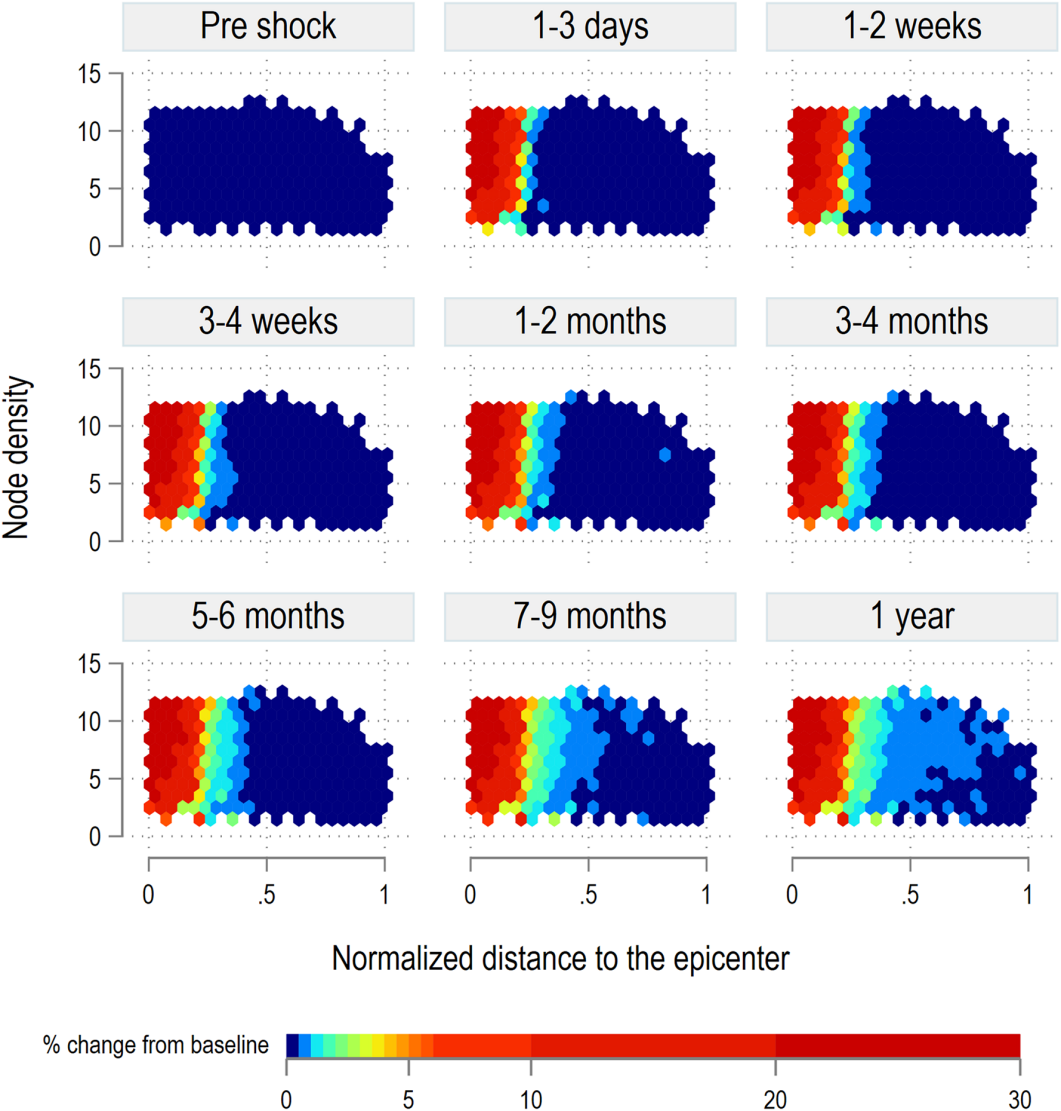
*VRank* percentage change.

Finally, Fig. 9 shows the results of the 80% food output shock on the distribution of vulnerability computed with the *VRank* index. We notice that the shock has a direct effect on the level of vulnerability of nodes, which steeply increases in particular for the nodes closest to the epicenter. In addition, once nodes becomes vulnerable, this condition is likely to persists even one year after the shock, when trade and migration flows have stabilized, and so the markets. The high-density nodes closest to the epicenter respond faster to the shock than the low- and mid-density ones. Again, higher density implies that the condition of vulnerability cascades to the neighboring nodes, where the response time of low-density nodes is much slower. We notice a stable post-shock distribution of the *VRank* in the last time steps implying that when the system stabilizes, nodes closer to the epicenter remain highly vulnerable while high density nodes also play a role in passing this vulnerability further away from the epicenter. Recovery dynamics are discussed in the Supplementary Appendix.

## Conclusions and research steps ahead

Natural disasters have significant socioeconomic impacts that spread beyond the epicenter of the shock. Indirect impacts are particularly relevant for agricultural, low-income regions because disaster shocks can impact both the supply side (for example, via disruption of supply networks) and the demand side (for example, via income losses and migration), eventually triggering cascading losses that exacerbate vulnerabilities. These outcomes emerge from the bottom-up behavioral interaction of locations nodes across different layers in a spatially-explicit network setting. The network setup, and the behavioral interaction of its various layers, plays a major role in determining the post-shock transition outcomes.

There is growing awareness of the fact that traditional economic modeling approaches and network models face limitations to assess the economic complexity of outcomes related to climate-led shocks. This requires the consideration of the underlying spatial-temporal distributional changes in a socioeconomic system that can give rise to system-wide vulnerabilities as well as the complexities in the behavioral response of the system. Identifying the shocks’ transmission channels, the drivers of cascading losses, and the socioeconomic nodes that are more vulnerable yet relevant, is crucial to inform timely and effective policies to build resilience.

To address this research challenge, we introduce a multi-layer behavioral network framework to analyze the short-term socioeconomic impacts of climate-led natural disasters on location nodes interconnected in a food trade network setting. Our framework allows us to embed heterogeneous spatial and temporal preferences, asymmetric information, and path-dependent post-shock outcomes. In addition, it allows us to adopt a modular approach for integrating heterogeneous behavioral response at the level of individual layer in the micro, meso, and macro level of analysis. Thus, our model framework is flexible enough to be further extended and tailored to specific case studies.

Our model contributes to the economic complexity literature, and to state-of-the-art disaster risk assessment by, (i) assessing the conditions for natural disasters to generate cascading losses via the multi-layer behavioral networks, (ii) identifying the patters of responses to shocks that are able to trigger a main change in socioeconomic vulnerability (via output, price volatility, income inequality), thus including the distributive effects, (iii) estimating the critical values for which the system reaches tipping points that trigger fundamental changes in behavior, and (iv), identifying the characteristics of resilient network structure that allows to mitigate cascading losses.

We apply the theoretical model to the case of a two-layer (supply-side production, and demand-side household) stylized economy that engages in food production and trade. In the model, location nodes in one part of a spatially-explicit multi-layer network are exposed to a natural disaster shock resulting in loss of food output. Simulations explore the distribution and evolution of vulnerability as the shock cascades across the network. These patterns are analyzed in a model which fully accommodates population displacement and supply-chain disruptions through market adjustments. In a real-world setting, where physical and institutional barriers can hinder a full market adjustment, for example through employment and trade restrictions, outcomes are likely to be worse. Then, we propose a new multi-layer measure, Vulnerability Rank or *VRank*, that synthesizes different risks associated with affording a minimum consumption bundle in a single index. The *VRank* index factors in prices, incomes, and trade and migration flows from neighboring nodes, and therefore also captures the network effects of vulnerability. Our results show that vulnerability is cyclical over time, and the distribution of vulnerability depend critically on the network density, and distance to the epicenter of the shock.

Our model, depending on the resolution of the study area, can be calibrated with actual data to support with policy planning. On a broader level, this could, for example, include information on regional trade and migration, and even social media or cell phone data^70^. Various publicly available datasets on natural disaster impacts include the EM-DAT, and the IDMC, and IOM databases on internal displacement and migration respectively. Several other databases can support this research. For example, the Atlas of Economic Complexity provides highly dis-aggregated product-level trade data^71^. Such a dataset could allow us to assess the role on food trade network shocks. Additionally, high-resolution satellite images like the Nightlight data, NASA Earth Observatory, and WorldPop Project allow for the mapping of spatial changes in populations and their related socioeconomic indicators. Similarly, the on-going COVID-19 crisis has also resulted in unprecedented amount of data being generated to track the pandemic^72^. The impact of lock-downs, border controls and mobility restrictions can also be translated into multi-layer networks that can be analyzed to estimate the direct and indirect impact of the pandemic.

A multi-layer complex network framework presented above can also be extended to study more advanced economies that are increasingly facing higher incidences of extreme climate shocks, a trend that is predicted to continue in the future as well^17^. For high-income regions, the multi-layer framework will need to also incorporate financial and public sector layers, in additional to trade and migration layers.

Another topic which can further evolve are multi-layer risk measures. Multi-layer networks are a relatively new field where recent efforts have focused on establishing new measures^67^ which range from quantifying network properties, generating a new set of indicators^24^ and defining new multi-layer risk measures^27,66,73^. These concepts can be easily ported network-based agent-based models and additional multi-layer risk measures can be easily established, for example, by incorporating recently proposed natural disaster-related resilience indicators^74^. The above innovations can provide a better understanding of post-disaster outcomes and can allow for nuanced policy responses.

## Supplementary Information

Supplementary Information.
