# Supplementary material for: Assessing the cascading impacts of natural disasters in a multi-layer behavioral network framework
Source: Sci Rep. 2021 Oct 11;11:20146. doi: 10.1038/s41598-021-99343-4 (PMC8505522; doi:10.1038/s41598-021-99343-4)

Assessing the cascading impacts of natural disasters in a multi-layer behavioral network framework

Asjad Naqvi Irene Monasterolo

Appendix

A Recovery

In this section, we discuss the situation where the affected regions are allowed to recover from the food production shock. Figure S1 shows the recovery pathway of two variables; average food consumption and food-to-labor relative price. Both the axis show the percentage change from the baseline no-shock scenario. Here we see that as food becomes more expensive, average consumption falls but also stabilizes as supply chains and populations adjust, reaching a stable point after the adjustment phase ends. As discussed earlier, the low points in the adjustment cycle help identify new vulnerabilities that are created in the post-shock transition phase. If recovery is allowed, the adjustment pathway follows a different trajectory. As food prices fall, average food consumption rises relatively linearly, till it reaches a peak and then stabilizes to some point around the individual starting point.

The two indicators shown in Figure S1 are plotted on the x-axis against *VRank* on the y-axis in Figure S2. The left figures shows that as food becomes relatively more expensive, *VRank* does not rise immediately, but shows an exponential increase towards the end. This exponential increase in *VRank* highlights that while some resilience helps the agents survive the initial phases of the shock through coping strategies, after some tipping point is crossed, these strategies are not sufficient to maintain even basic consumption levels. In the recovery phase, the decline in *VRank* mirrors the post-shock phase. In contrast, *VRank* and average food consumption have an unusual relationship. As average consumption goes down, *VRank* stays fairly stable since the system has enough food to cater in the initial adjustment phases. Over time as populations move away from the epicenter of the shock and supply chains adjust, *VRank* explodes resulting from a high stress in the system. Here we can also see in

Figure S1: The recovery cycle

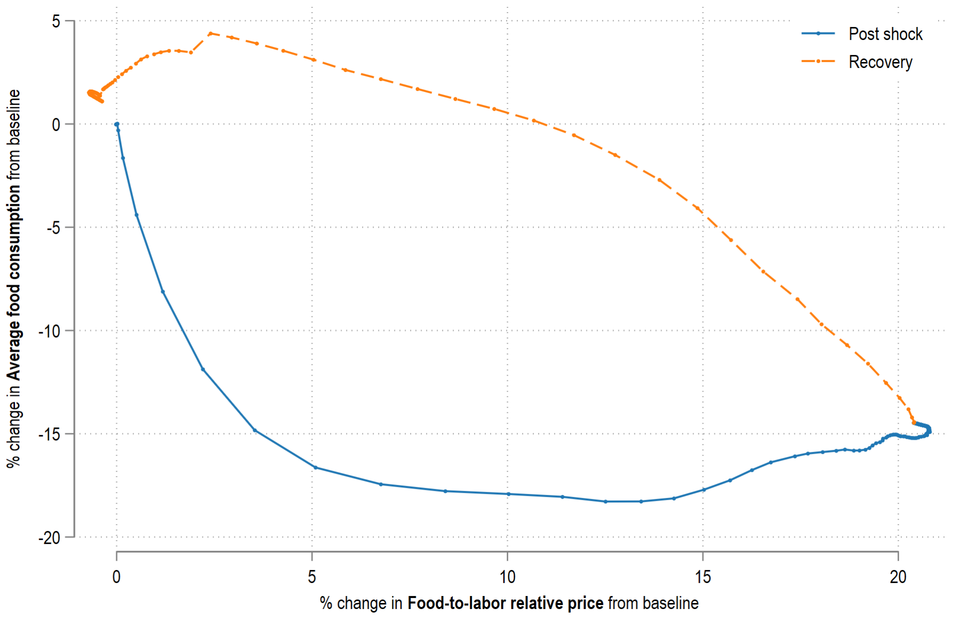

Note: Adjustment takes place in a counter-clockwise cycle.

the recovery phase that as food slowly becomes available, the fall in *VRank* is gradual and only after a certain threshold, it drops significantly.

Figure S2: The *VRank* recovery cycle

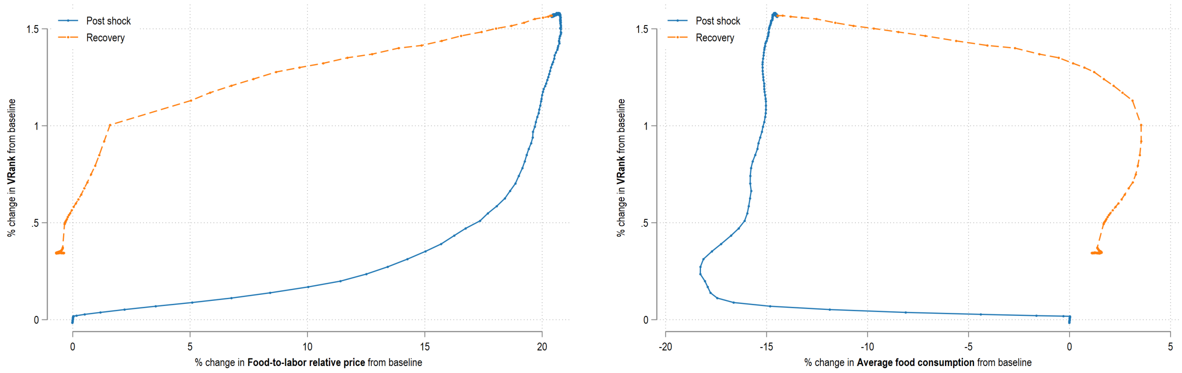

(a) (b)

Figure S3 plots the distributional changes in the post-shock and the recovery phase. Here

the first nine figures represent the post-shock adjustment phase (similar to the ones in the main text above), while the next nine show the recovery phase. Following from the earlier graphs, x-axis represents normalized distance to the epicenter while the y-axis represents node density. The recovery of the average food consumption is as expected. As the affected regions are re-built, and food again becomes available all locations see an increase in food consumption. Higher density nodes allow for faster recovery due to higher connectivity. In terms of relative food to labor prices, the adjustment is slower than consumption levels. Here we can also observe higher frictions especially in low density markets taking longer to return to normal.

Figure S4 looks at the distributional impacts of the recovery on *VRank*. Here the affected regions have the highest stress levels which is quickly passed on to neighboring regions and this slowly cascades away from the epicenter. In the recovery phase, the higher vulnerability tends to stay in place where higher density nodes return to pre-shock trends faster, indicating a higher level of resilience. After the price and consumption levels converge and the system stabilizes, vulnerability still persists and stays slightly higher that the baseline levels. This implies that even rebuilding back to baseline does not fully alleviate the vulnerabilities induced during the post-shock phase and potentially require additional interventions for a full recovery.

Figure S3: Distributional impacts of the recovery

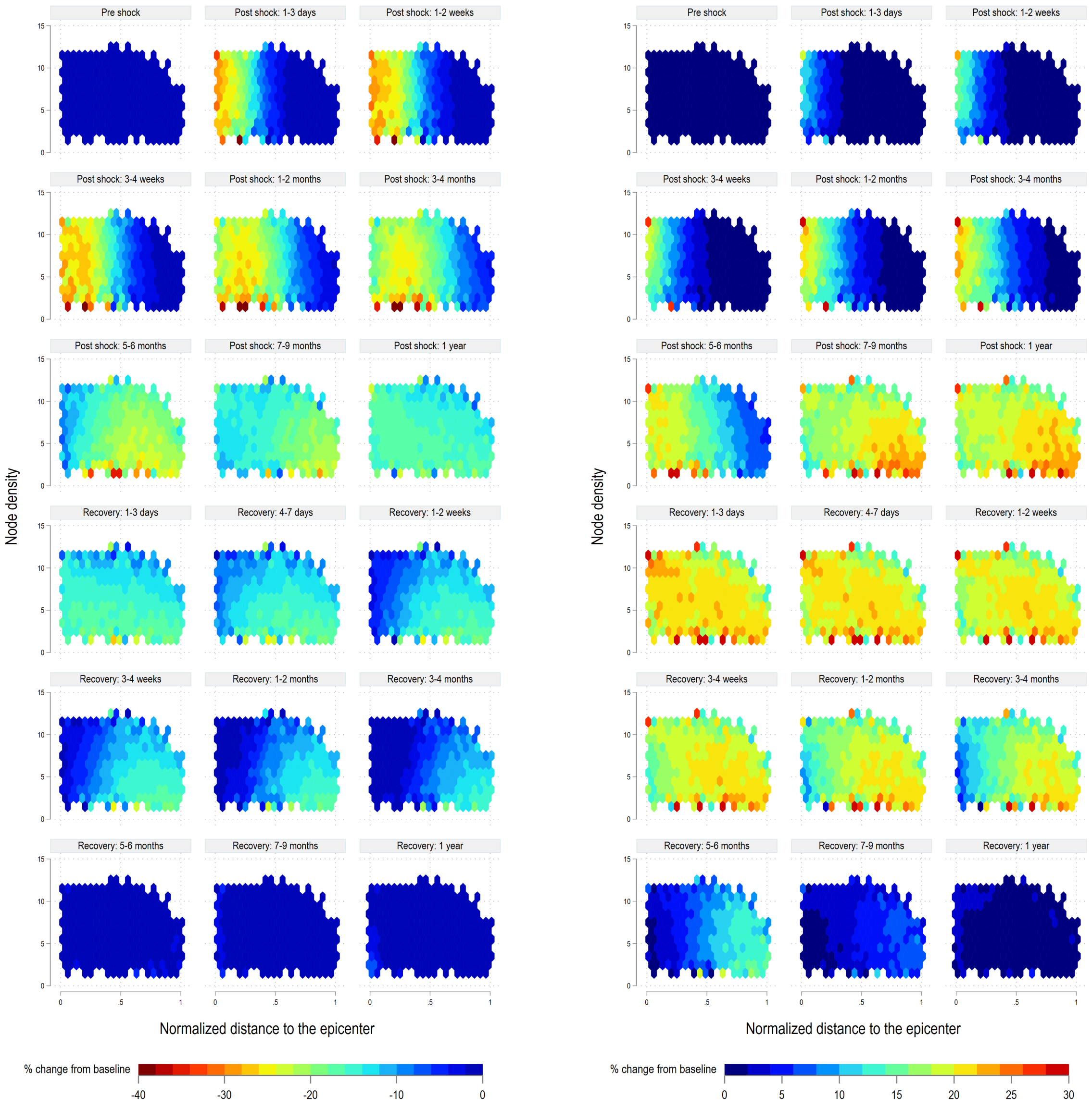

(a) Average food consumption

(b) Food-to-labor price ratio

Figure S4: Distributional impacts of recovery on *VRank*

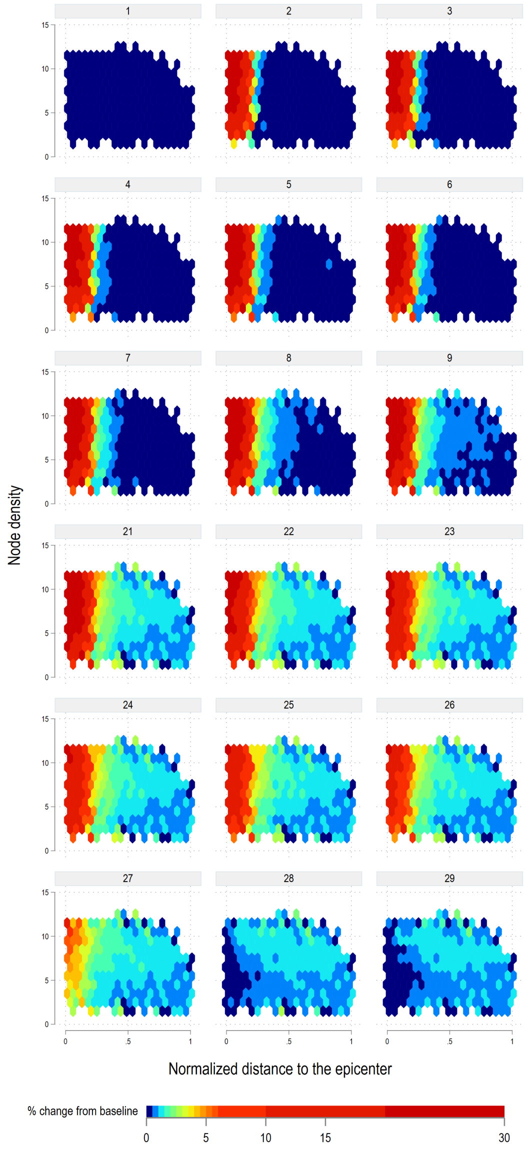

Supplement: Supplementary file 1 — Supplementary Information. [file 41598_2021_99343_MOESM1_ESM.pdf]
